# Complement enhances in vitro neutralizing potency of antibodies to human cytomegalovirus glycoprotein B (gB) and immune sera induced by gB/MF59 vaccination

**DOI:** 10.1038/s41541-017-0038-0

**Published:** 2017-12-14

**Authors:** Fengsheng Li, Daniel C. Freed, Aimin Tang, Richard R. Rustandi, Matthew C. Troutman, Amy S. Espeseth, Ningyan Zhang, Zhiqiang An, Michael McVoy, Hua Zhu, Sha Ha, Dai Wang, Stuart P. Adler, Tong-Ming Fu

**Affiliations:** 1 Department of Vaccines Research, MRL, Merck & Co., Inc, Kenilworth, NJ USA; 2 Texas Therapeutics Institute, Brown Foundation Institute of Molecular Medicine, University of Texas Health Science Center at Houston, Houston, TX 77030 USA; 3 Virginia Commonwealth University, Richmond, VA USA; 4 Rutgers New Jersey Medical School, Newark, NJ USA; 5 CMV Research Foundation, Richmond, VA USA

## Abstract

Human cytomegalovirus (HCMV) is the leading cause of in utero viral infection in the United States. Since congenital HCMV infection can lead to birth defects in newborns, developing a prophylactic vaccine is a high priority. One of the early experimental vaccines, composed of a recombinant glycoprotein B (gB) formulated with MF59 adjuvant, has demonstrated approximately 50% efficacy against HCMV infection in seronegative women. Using immune sera from two gB/MF59 Phase 1 studies in humans we showed that complement can enhance the in vitro HCMV neutralizing potency of antibodies induced by the gB/MF59 vaccination. To characterize this complement-dependent antiviral activity, we analyzed three rabbit non-neutralizing gB monoclonal antibodies (mAbs) with different biochemical profiles including epitope specificity. Two of the three mAbs, r272.7 and r210.4, exhibited neutralizing activity when complement was added to the assays, and this complement-dependent antiviral activity was not related to the antibody’s affinity to gB but appeared to be associated with their epitope specificities. Moreover, neutralization could only be demonstrated when complement was present at or before viral entry, suggesting that IgG Fc-mediated function was not the basis for this antiviral activity. Lastly, we demonstrated that gB/MF59 immune sera contained antibodies that can cross-compete with r272.7 for gB binding and that the titers of these antibodies correlated with complement-dependent neutralization titers. These results suggested that gB antibodies with certain biochemical properties have neutralizing potency when complement is present and that this complement-dependent antiviral activity may be a part of immune components which conferred protection against HCMV infection by gB/MF59 vaccination.

## Fetal infection: Complementing vaccine performance

Enhancing a cytomegalovirus vaccine candidate with immune system proteins could be a step closer to protection against pre-birth infections. Cytomegalovirus is the leading cause of *in utero* infections in the United States; however, the leading vaccine candidate against the virus is, at best, 50% effective. A US group of scientists led by Merck’s Tong-Ming Fu found that adding a set of innate, antipathogenic proteins called ‘complement’ to the candidate increased the protective effects of immunization in humans, and *in vitro* with a selection of otherwise non-protective antibodies. The team also found that complement augmented the vaccine through a non-standard mechanism, which was dependent on the specific profile of the antibodies fighting the infection. This article describes an interesting mechanism of vaccine protection that warrants further investigation.

## Introduction

Human cytomegalovirus (HCMV) is a common β-herpesvirus which rarely causes any discernible disease in healthy children and adults; however, in utero infection of HCMV or infection in immune-compromised patients can lead to severe consequences. Congenital HCMV infection is the leading cause of non-genetic birth defects in the United States.^1,2^ It is estimated that over 5500 newborns suffer from sequelae of congenital HCMV infection each year, with clinical manifestations including microcephaly, sensorineural hearing and/or vision loss, intellectual disability and psychomotor impairment.^2^ Developing a prophylactic vaccine against congenital HCMV infection and disease has been assigned to the category of top priority by the Institute of Medicine.^3^ In immune-compromised individuals such as those under immunosuppression post stem-cell or solid-organ transplantation, HCMV is the most frequently encountered infectious pathogen, despite the routine use of antiviral small molecule drugs in clinical practice.^4^ Restoration or reconstitution of host anti-HCMV immunity could provide long-term control of HCMV post transplantation.^5,6^ However, despite tremendous unmet medical needs and active vaccine research in the past 40 years, there is still no approved vaccine.^7,8^

Vaccine candidates for prevention of congenital HCMV infection generally fall into two categories^8^: those composed of modified whole viruses, such as the live attenuated virus Towne vaccine,^9^ and those focusing on individual viral antigens, exemplified by the recombinant glycoprotein B (gB) vaccine formulated with an oil-in-water emulsion adjuvant MF59 (gB/MF59).^10^ Towne and gB/MF59 vaccines are the most advanced candidates in development; both have been tested in several Phase 2 efficacy trials, and the results are informative for current research efforts on vaccine design and characterization. The Towne vaccine failed to protect HCMV seronegative women against acquisition of wild-type virus from their young children in daycare.^11^ In addition, it did not provide protection against HCMV infection in renal transplant recipients although it was effective against severe HCMV disease.^7,12^ Lastly, the Towne vaccine provided protection against viral challenge with a low passage pathogenic Toledo strain in HCMV seronegative vaccine recipients, however, the protection was less effective when compared to the immunity conferred by natural infection in HCMV seropositive subjects.^13^ These trials collectively suggest that there are protective components in the immune responses by Towne vaccination. However, the large number of antigens present in the Towne vaccine makes it challenging to determine which antigen components or what types of immune responses are important for the observed protection. In contrast, the gB/MF59 vaccine is composed of a single truncated viral glycoprotein, and its design goal is to induce antiviral antibodies. The gB/MF59 vaccine has been evaluated in efficacy trials for prevention of HCMV acquisition in HCMV seronegative women or adolescent girls, and for prevention of HCMV viremia in solid-organ transplant recipients. Overall, the vaccine achieved approximately 50% efficacy for protection against HCMV acquisition in seronegative women, mostly in the first 12 months post vaccination.^14,15^ It was also effective in reducing viral episodes in both HCMV seropositive and seronegative transplant recipients.^16^ Thus, further characterization of the gB/MF59 immune responses may lead to better understanding of the immune response required to prevent HCMV infection.

We had reported that the immune sera from rabbits immunized with a recombinant gB formulated with an oil-in-water emulsion adjuvant failed to neutralize virus in either MRC-5 or ARPE-19 cells; however, we could observe neutralizing activities when rabbit complement was supplemented in the neutralization assays.^17^ In addition, our previous work has identified a panel of rabbit monoclonal antibodies (mAbs) to HCMV, and all mAbs specific to gB in this panel lack neutralizing activity in cell culture assays.^18^ To understand this complement-dependent neutralization, we selected three non-neutralizing gB mAbs with different biochemical profiles for further analysis. Here we show that two of the three gB mAb can neutralize HCMV when rabbit complement was supplemented in the assays. Furthermore, the neutralizing activities of the human immune sera from previous gB/MF59 vaccine trials were enhanced by complement. Our results suggest that complement-enhanced neutralization by antibodies induced by gB/MF59 vaccination may play a role in antiviral activity which contributed to the efficacy observed against HCMV infection in seronegative women.

## Results

### Complement is required for neutralizing activity of some gB-specific mAbs

**Fig. 1:**
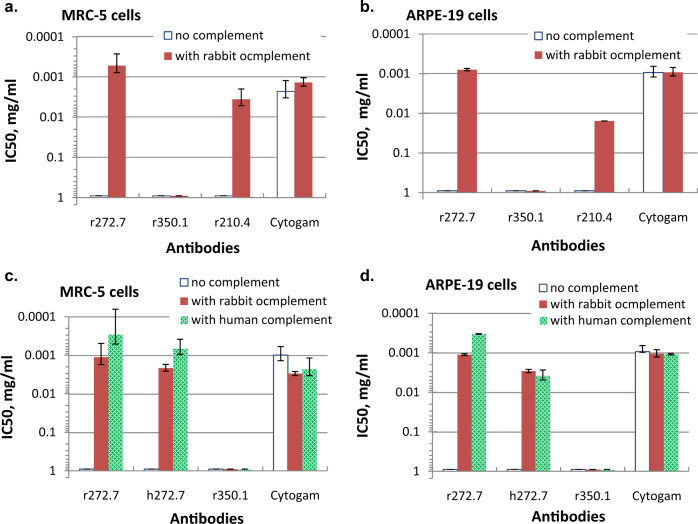
Complement-dependent neutralization by selected gB mAbs in MRC-5 and ARPE-19 cells. Three rabbit mAbs or CytoGam^®^ were mixed with HCMV (AD169rev-GFP) with or without rabbit complement. After incubation for 1 h at 37 °C, the mixture was added to MRC-5 (**a**) or ARPE-19 cells (**b**). Antibody r272.7 and its humanized version h272.7, along with r350.1 and CytoGam^®^, were tested for neutralization with no complement, or with rabbit complement or human complement in MRC-5 (**c**) or ARPE-19 (**d**) cells. IC_50_ values, defined as IgG concentrations required to neutralize 50% of viral infectivity were calculated by four-parameter curve fitting. Rabbit complement was added at 1:64 final dilution and human complement at 1:32. Human complement was sourced from a healthy donor of HCMV seronegative status. The results shown are representative of two independent experiments. The error bars represent the standard deviations.

Three gB-specific rabbit mAbs, all IgGκ lacking neutralizing activity in vitro, were evaluated for complement-dependent neutralization of HCMV.^18^ With no complement, none of the three antibodies had neutralizing activity in either MRC-5 or ARPE-19 cells, in contrast to CytoGam^®^, human CMV hyperimmune globulin (Fig. 1). When rabbit complement was added to the assays, antibodies r272.7 and r210.4, but not r350.1, exhibited neutralizing activity in both cell types. The neutralization potency was calculated by the concentration of IgG needed to achieve 50% inhibition of viral infection (IC_50_), and the IC_50_ values for r272.7 were 0.5 and 0.8 μg/mL in MRC-5 and APRE-19 cells, respectively, slightly better than those of CytoGam^®^ (Fig. 1a, b). Rabbit complement did not enhance the activity of CytoGam^®^, and without complement, its IC_50_ value in MRC-5 cells was about 3-fold lower when compared with that in ARPE-19 cells, consistent with previous reports.^19,20^ To address the possibility that antibody affinity played a role in this complement-dependent antiviral activity, we measured the relative affinity to gB for each antibody, and the affinity was quantified by the antibody concentration required to generate 50% of maximal binding signal in enzyme-linked immunosorbent assay (ELISA; EC_50_).^18^ The EC_50_ values for mAbs r272.7, r350.1 and r210.4 were measured as 0.002, 0.031 and 0.017 μg/mL, respectively (Supplementary Fig. S1). Thus, although r350.1 and r210.4 had similar affinity to gB in ELISA, their neutralizing profile with complement were different. In conclusion, gB-specific mAbs r272.2 and r210.4, previously described as non-neutralizing, exhibited neutralization activity when complement was added, and this complement-dependent antiviral activity appeared unrelated to their affinity to gB.

Next, we evaluated kinetics of the complement effect on neutralization by gB-specific mAbs. The complement-dependent neutralization could only be observed when the complement was added to the mixture of mAb r272.7 and HCMV in suspension prior to applying to the MRC-5 or ARPE-19 cell monolayers; no virus neutralization was observed if complement was added to cell monolayers 1 h after adding the mAb r272.7 and virus mixture to the cells (data not shown). In addition, there was no sign of cytotoxic effects when mAb r272.7 and complement were added to cells 24 h after viral infection, and there was no increase of lactate dehydrogenase in the culture supernatant above the baseline, an indication of the membrane damage of the virus-infected cells at 24 h post infection (data not shown). Thus, this gB-specific antibody-mediated neutralization seemed dependent on the presence of complement prior to viral attachment/entry.

### Both rabbit and human complement enhance neutralization by gB-specific antibodies

To evaluate the complement effects with human immune sera, we wanted to assess if there was species restriction on the source of complement. We first humanized mAb r272.7 (Meng et al., in press for publication in *Antimicrobial Agents and Chemotherapy*), and the new antibody, named h272.7, demonstrated approximately the same EC_50_ value of 0.002 μg/mL as parental antibody r272.7. We then tested HCMV neutralization using fresh serum from a donor of HCMV seronegative status as a source of human complement. The neutralization was evaluated for the effects of human vs. rabbit complement on h272.7 vs. parental r272.7; CytoGam^®^ and mAb r350.1 were included as positive and negative controls, respectively (Fig. 1c, d). Rabbit complement was equally effective in mediating neutralizing activity of r272.7 and h272.7, with both conditions producing IC_50_ values of 1.1 and 2.1 μg/mL, respectively in MRC-5 cells, and 1.1 and 2.9 μg/mL, respectively in ARPE-19 cells. Human complement did not show any better efficiency in mediating neutralization of h272.7 than r272.7, as the IC_50_ for r272.7 with human complement were lower than those of h272.7 in MRC-5 and ARPE-19 cells. As expected, mAb r350.1 showed no activity with either source of complement, and the neutralizing potency of CytoGam^®^ was not significantly affected by either type of complement. These results suggested that rabbit and human complement were equally effective in enhancing neutralizing activity of the gB antibodies of either human or rabbit IgG Fc region; rabbit complement can be used to assess human antibodies to gB in neutralization assays.

### Characterization of binding profiles of gB-specific mAbs

**Fig. 2:**
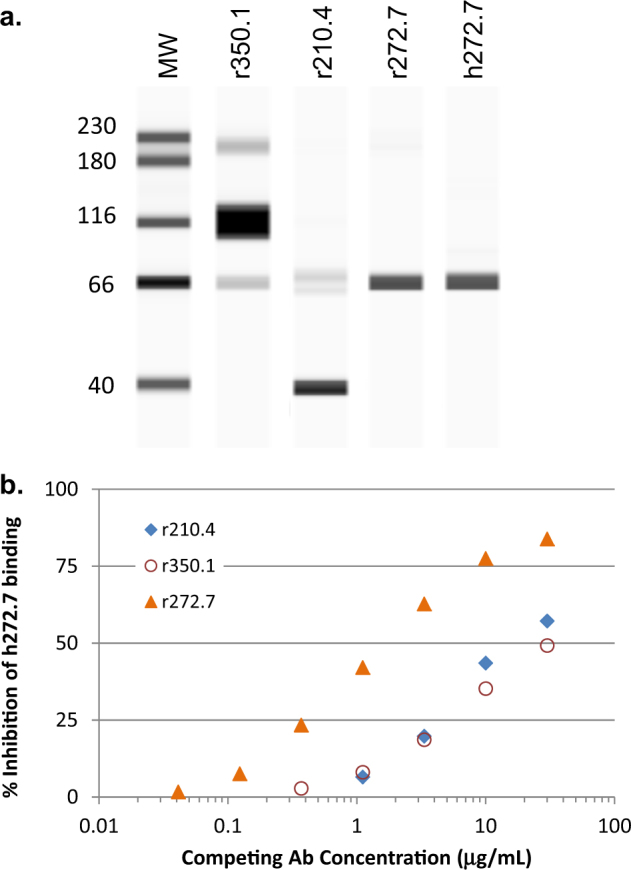
Biochemical property of r272.7 is  different from that of r350.1 and r240.4. HCMV virus (AD169rev-GFP) was denatured and reduced for antibody detection in Western blot analysis (**a**). The full-length gel blot is shown in Supplementary Fig. S4, and sample derived from the same experiment and gel blot were processed in parallel. Three rabbit antibodies in titration were tested to compete with h272.7 at a fixed concentration in ELISA (**b**). The results are representative of two experiments

Full-length gB is cleaved by furin into a 116-kDa N-terminal and a 55-kDa C-terminal subunits.^21^ Western analyses using denatured and reduced HCMV virions revealed that mAb r350.1 recognized a 116-kDa protein likely representing the N-terminal subunit of gB; both r210.4 and r272.7 recognized a 55-kDa that presumably represents the C-terminal subunit of gB, and as expected, h272.7 exhibited the same recognition pattern as r272.7 (Fig. 2a). However, unlike r272.7, mAb r210.4 also reacted strongly to an approximately 40 kDa protein, the nature of which has not been resolved. These results suggested that all three antibodies likely react with different epitopes on gB, with the epitope for r350.1 located in N-terminal subunit and the epitopes for r272.7 and mAb r210.4 located in the C-terminal subunit.

To further support this conclusion, mAb h272.7 was used to compete the gB binding of all three rabbit mAbs. As shown in Fig. 2b, r350.1 and r210.4 were not as effective in competing against mAb h272.7 binding to gB in ELISA as r272.7, thus confirming that mAb r272.7 recognized an epitope different from those of r210.4 and r350.1.

We attempted to map their epitopes using a peptide array composed of synthetic peptides of 15-mer in length overlapping by 11 amino acids, however, none of the three mAbs reacted to any of the linear gB peptides (data not shown). This data suggested that their epitopes may not be strictly linear but may depend on additional sequences or some degree of refolding that survived denaturation in Western blot. Alternatively, these mAbs may recognize epitopes with some post-translational modification such as glycosylation.

### Complement-dependent neutralizing antibodies induced by the gB/MF59 vaccine

The immune sera from two Phase 1 gB/MF59 trials were available for analysis; one trial was designed to test vaccine immunogenicity in a dose escalation scheme at 5, 30 and 100 μg/dose, and the other to evaluate the vaccine immunogenicity at 20 μg/dose. In both trials, gB/MF59 was administrated at study months 0, 1 and 6 as previously reported.^10^

**Fig. 3:**
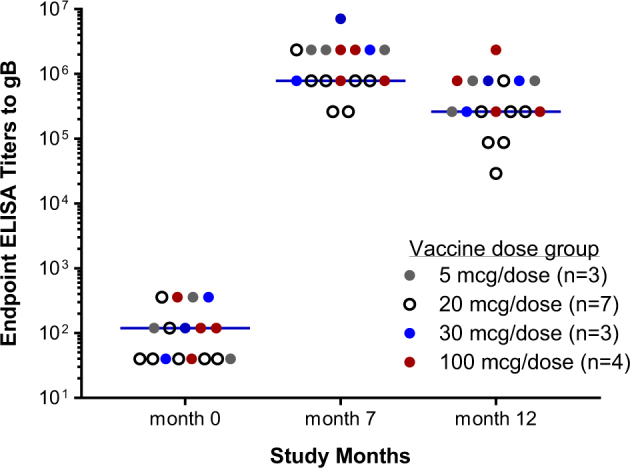
Dose responses of human subjects to the gB/MF59 vaccine. Cohorts received three immunizations of the gB/MF59 vaccine at the indicated doses at months 0, 1, and 6. Endpoint titers of gB-specific IgG were determined by ELISA. Each circle represents an individual serum sample and the colors of the circles represent the different dose levels as indicated in figure legend. The numbers of subjects at each dose level are shown in figure legend. Lines represent the geometric means for all samples from each time point

We first evaluated gB/MF59 immune sera for the reactivity to recombinant gB in ELISA, and the results for samples at the selected study month confirmed that the vaccine was highly immunogenic in HCMV seronegative human subjects (Fig. 3). All 17 subjects responded to vaccination and developed high levels of serum antibodies to gB, but no clear dose-response was observed among these vaccine subjects, consistent with an earlier report.^22^ To increase the power of analysis, we grouped all samples together regardless of the vaccine dose levels. The geometric mean titer (GMT) for all subjects prior to immunization (study month 0) was slightly above 100, and the GMT at 1 month after dose 3 (study month 7) was approaching 1 × 10^6^. The antibody levels persisted with the GMT maintained at 3.4 × 10^5^ at 6 month after dose 3 (study month 12).

**Fig. 4:**
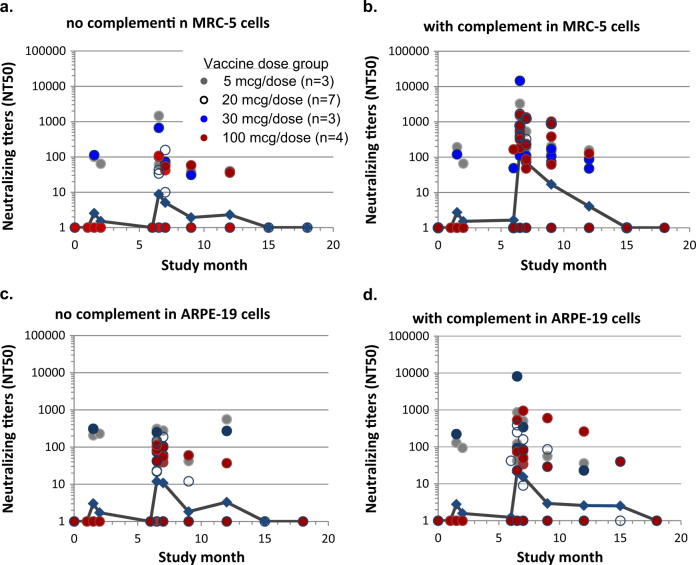
Longitudinal neutralizing activities induced by the gB/MF59 vaccine. The neutralizing titers of immune sera from subjects with the gB/MF59 vaccination were determined with no **a**, **c** or with **b**, **d** complement in assays in MRC-5 (**a**, **b**) or ARPE-19 (**c**, **d**) cells. Neutralizing titers (NT_50_) were calculated on reciprocal serum dilution to achieve 50% viral neutralization by four parameter curve fitting. If there is no neutralizing activity or poor curve fitting, an NT_50_ titer of 1 was assigned. Each circle represents one serum sample and the colors of the circles represent the different dose levels as indicated in figure legend. The numbers of subjects at each dose level are shown in figure legend. Blue lines connect the geometric means of all serum samples at each time point, regardless of the vaccine dose levels

The gB/MF59 immune sera were then tested for neutralizing activities with or without rabbit complement against HCMV infection in MRC-5 and ARPE-19 cells. The neutralizing titers were calculated as the reciprocal of serum dilutions resulting in neutralization of 50% input virus (NT_50_). NT_50_ titers with or without complement were plotted longitudinally. Only three out of 10 subjects developed neutralizing antibodies in MRC-5 cells, regardless of complement, after the first and second immunization (Fig. 4a, b). After the third immunization high neutralizing titers were observed in 14 out of 17 subjects but these activities were highly complement-dependent. Two weeks after dose 3 the GMT of the neutralizing titers in MRC-5 cells was 160 with complement vs. 9 without complement. Similar complement-dependent neutralizing titers were sustained at study months 7 and 9 (1 and 3 months after the third dose) and remained detectable in four out of 17 subjects at study month 12. The gB/MF59 vaccine was much less effective at eliciting neutralizing antibodies against viral epithelial cell entry (Fig. 4c, d), as only ten out of 17 subjects developed measurable epithelial entry neutralizing titers with complement at two weeks post dose 3 (study month 6.5), consistent with a previous report.^19^

To calculate the extent of complement dependence for the gB/MF59 immune serum, the neutralizing titers for individual serum samples obtained at study month 6.5 or 7 were paired for comparison analysis (Supplementary Fig. S2). The neutralizing GMTs with or without complement in MRC-5 cells were 108 and 7, respectively, or a 15-fold increase. This difference was statistically significant in a paired two-tailed *t*-test (*P* = 0.045). In contrast, the neutralizing GMTs in APRE-19 cells with or without complement were 18 and 11, respectively, or merely a 1.6-fold increase, with no statistical significance (*P* = 0.188).

Lastly, we calculated the responder rate for those vaccine recipients who developed NT_50_ ≥ 100. Complement changed the responder rates with titers against viral fibroblast entry from 20 to 78% at study month 6.5, and the responder rate sustained at 59, 41, and 15% at study months 7, 9, and 12, respectively. Complement did not significantly impact the responder rates for neutralizing titers against viral epithelial cell entry, as without complement the rates were 28 and 29% at study months 6.5 and 7, respectively, compared to rates of 33 and 24% with complement at the same time points.

### Association of r272.7-like antibodies in gB/MF59 sera with the complement-enhanced neutralizing activity

**Fig. 5:**
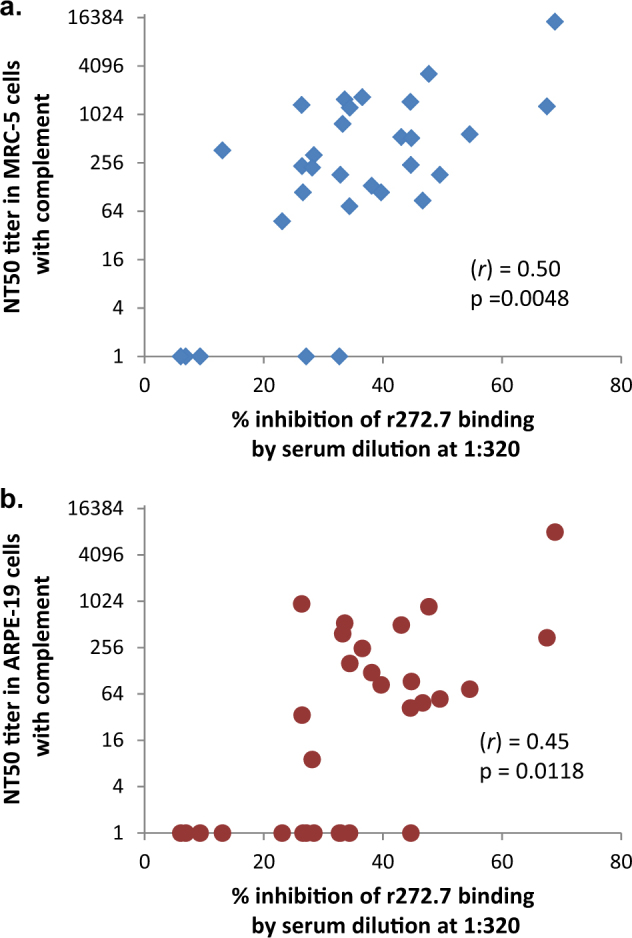
Correlation of complement-enhanced neutralizing titers with r272.7-like antibodies in the gB/MF59 immune sera. Immune sera obtain at study months 6.5 or 7 were measured for their ability to compete against r272.7 for binding to gB protein in ELISA. The percent inhibition of r272.7 binding at a fixed serum dilution of 1:320 was plotted vs. NT_50_ titers measured with complement using MRC-5 (**a**) or ARPE-19 (**b**) cells. (r) represents Pearson correlation coefficient

The mAb r272.7 exhibited the potent neutralizing activity with complement, so we next measured whether gB/MF59 immune sera contained antibodies that could cross-compete with r272.7 for binding to gB in competition ELISA. When tested at fixed serum dilution of 1:320, some of gB/MF59 immune sera inhibited r272.2 ELISA binding by up to 70%. To determine whether the presence of antibodies capable of competing against r272.2 for binding would correlate with complement-dependent serum neutralizing activities, NT_50_ titers determined with complement were plotted against percent inhibition of gB binding by r272.7 (Fig. 5). The positive correlation in both cases was demonstrated as the Pearson correlation coefficient (*r*) was 0.50 and 0.45 in MRC-5 and APRE-19 cells, respectively. The analyses also revealed *p* values of 0.0048 for the correlation in MRC-5 cells and 0.0118 in ARPE-19 cells, respectively. When the same analysis was performed using NT_50_ titers without complement (Supplementary Fig. S3), the correlation was not significant (Pearson (*r*)=0.36, *P* = 0.052 for the association in MRC-5 cells; Pearson (*r*)=0.29, *P* = 0.81 in ARPE-19 cells). These results indicated that when neutralizing activity in the gB/MF59 immune sera was measured using complement, the results positively correlated with the amount of antibodies in the sera that likely bound at or near the r272.7 epitope, such that their binding could interfere the r272.7 binding.

## Discussion

HCMV gB is considered an important viral antigen in vaccine compositions based on the observations that gB is essential in viral replication, serving as a viral fusogenic protein critical for viral entry into host cells, and also that prominent immune responses to gB are routinely detected in HCMV seropositive subjects.^23–25^ Supporting this notion, the experimental gB/MF59 vaccine is highly immunogenic in HCMV seronegative subjects, and importantly, it is effective against HCMV acquisition in young women.^14,15^ However, the protection conferred by the vaccination against HCMV infection was modest with approximately 50% efficacy, and was mostly observed during the first 12 months.^14^ Paradoxically, low neutralizing titers against viral entry in fibroblast and epithelial cells were detected in the immune sera of gB/MF59 vaccine recipients as compared to those of HCMV seropositive subjects.^16,19^ Thus, the efficacy seen from gB/MF59 vaccination has been speculated from effector functions such as antibody-dependent cell-mediated cytotoxicity or complement-dependent cytotoxicity (CDC) mediated through IgG Fc. The results presented here offered an alternative explanation to the observed efficacy by gB/MF59 vaccination. With a panel of gB mAbs with no neutralizing activity, we showed that the addition of complement can change their antiviral property in neutralization assays in vitro. Two mAbs r272.7 and r210.4 exhibited neutralizing activity when complement was present in the assays. With complement, mAb r272.7 demonstrated potent neutralization in both MRC-5 and ARPE-19 cells with IC_50_ values comparable to those of CytoGam^®^. Intriguingly, this complement-dependent antiviral function seemed not related to antibodies’ affinity to gB, but likely associated with the epitopes they recognize.

The antiviral mechanism for complement is commonly explained by the antibodies recognizing viral antigens on the infected cell surface or virion envelope, and this would in turn trigger complement activation cascades which assemble the complement complex leading to membrane damage, known as CDC or virolysis.^26–28^ However, this mechanism could not explain our observation. First, although all three antibodies were the same isotype of IgGκ, the complement-mediated antiviral activity was not demonstrated for all three, suggesting that binding to gB and the ability to fix complement was not sufficient for the observed neutralization in culture. Second, the antiviral activity could only be observed when complement was added with mAbs to virus prior to applying the mixture to cell monolayers; no neutralization was observed if the complement and antibody mixture was added 1 h or 24 h post viral infection. The result suggested that, in this particular case, the complement-mediated antiviral function was mostly effective at targeting non-cell-associated virions in suspension, prior to their attachment to cell surface. Also, there was no cytotoxicity detected with complement in viral neutralization assay that could be suggestive of any antiviral activity targeting virus-infected cells.

There are many gB-specific mAbs that have been described. Although the majority of these mAbs demonstrate poor antiviral activity in cell culture,^18,29–31^ there are neutralizing antibodies with activity independent of complement, with some of these mapped to gB antigenic determinant (AD)-2, AD-4 and AD-5 regions.^30–33^ There are also several gB-specific mAbs that have been described as having neutralizing activity that is complement-dependent.^34–36^ In addition, complement-enhancing neutralization without virolysis has been described, and one proposed mechanism for this is that the accumulation of complement on viral envelop would inhibit viral interaction with its cellular receptor required for viral entry.^28,37^ Our data were consistent with this proposed mechanism, and our results further indicated that this mechanism could be affected by the antibodies’ biochemical properties, including epitope specificity. The antibodies in our study exhibited different neutralizing profiles in the presence of complement and different patterns in Western blot analysis. These observations suggested that their antiviral activity with complement was likely associated with their epitope specificity. Since we could not identify the epitopes of these mAbs, the exact mechanism underlying their complement-dependent antiviral activity remains to be determined.

That complement had a much larger impact on the neutralizing activity of gB/MF59 immune sera against viral fibroblast entry vs. epithelial entry suggests that viral epithelial entry is less sensitive to antiviral antibodies targeting these complement-dependent neutralizing epitopes. Similar data have been reported for sera from rabbits immunized with gB-encoding DNA vaccine.^38^ However, this phenomenon with the immune sera was not observed with mAb r272.7 which displayed similar neutralizing potencies of blocking viral fibroblast vs. epithelial entry. This difference in mAbs vs. the immune sera could be explained as the gB/MF59 immune sera may contain antibodies to additional complement-dependent neutralizing epitopes in gB, such as the one of r210.4. The r210.4 IC_50_ values with complement were determined as 3.6 vs. 15.5 μg/mL, respectively in MRC-5 cells and ARPE-19 cells, about 5-fold difference in its potency against viral fibroblast vs. epithelial entry (Fig. 1a, b). Thus, the inhibition seemed to be related with antibody’s epitope specificity as the epitopes defined by r272.7 and r210.4, but certain regions of gB may be more selective at blocking viral fibroblast over epithelial entry as the epitope defined by r210.4. Future efforts to define these antibodies’ and other mAbs’ epitopes may offer important clues to the underlying mechanisms.

The presence of r272.7-like antibodies in the gB/MF59 immune sera was demonstrated by the competition assays. Correlation between the levels of r272.7-competing antibodies and complement-enhanced neutralizing activity suggested that antibodies targeting the r272.7 epitope may be in part responsible for the complement dependence of gB/MF59 immune sera, and given that complement is abundantly present in vivo, complement-dependent neutralizing activities could be a contributor toward the observed clinical efficacy of the gB/MF59 vaccine.^14,15^

There are several interesting observations. First, the effects of complement-enhanced neutralization were more pronounced in MRC-5 cells, reflected not only in the higher neutralizing titers, but also the higher percent responders post vaccination. This was inconsistent with the comparable IC_50_ values in MRC-5 and ARPE-19 cells that we observed for r272.7 with complement in neutralization assays, and it could suggest that additional antibodies or mechanism would be also be in play for the observed antiviral activity with complement. Second, complement-enhancing effects were mostly observed after third immunization at study month 6, indicating that the antiviral activities were mediated through gB-specific IgG, not IgM. Lastly, the complement-dependent neutralization were not durable with most of the antiviral activities observed within 6 months post the third vaccination; this result could in part explain the short duration for the observed efficacy against HCMV acquisition after gB/MF59 vaccination.^14^ However, it should be noted that the gB/MF59 immune sera used in our study were from two Phase 1 safety and immunogenicity trials, not those of the Phase 2 efficacy trials. With the serum samples analyzed in this study, it would not be possible to definitely link r272.7-competing antibodies to the reported efficacy in those clinical trials, and based on the current data, it would be challenging to determine whether the r272.7-like antibody titer be a useful surrogate for future vaccine studies.

In summary, we described a complement-dependent neutralization with gB-specific antibodies, and such antiviral activity appeared to be associated with their biochemical profiles, including epitope specificities, not with their affinity to gB, as shown for some mAbs. Complement-enhanced neutralization was also demonstrated in the human gB/MF59 immune sera, and this activity correlated with antibodies in the immune sera that could compete for binding with mAb r272.7. These results suggested that the enhanced neutralization by complement may be a part of the immune components that confer protection against HCMV infection by gB/MF59 vaccination.

## Materials and methods

### Reagents, cells, virus, mAbs and human sera

The gB protein was based on Towne strain sequence as described previously.^39^ The protein was purified from HEK293 cells using transient transfection (Sinobiologicals, Inc., Beijing, China). MRC-5 and ARPE-19 cells, and AD169 and AD169rev viruses were cultured as previously described.^20^ AD169rev-GFP virus was a generous gift of Thomas Shenk of Princeton University. Rabbit complement was purchased from Cedarlane (Burlington, Ontario, Canada). Human complement was obtained from fresh serum of a HCMV seronegative human donor with informed consent and the collection was approved by Merck internal ethic review committee. Rabbit mAbs were isolated from a rabbit immunized with AD169rev  vaccination, and the three mAbs specific to gB were selected based on their unique biochemical properties.^18^ CytoGam^®^, lot#905332, was manufactured by CSL Behring LLC. Human immune sera were collected from study subjects with informed consent in clinical trials of the gB/MF 59 vaccine as previously described.^19,22,40^ Methods were performed in accordance with relevant regulations and guidelines. Methods and study protocols were approved by Institutional Review Board of Virginia Commonwealth University, Richmond, VA. Human sera were inactivated at 56 ^o^C for 30 min prior to in vitro assays.

### Western blot analysis

Purified HCMV virions (AD169) were denatured and reduced, and used as antigen source in capillary Western, Wes^™^ (ProteinSimple, Santa Clara, CA) as previously described.^41^ Briefly, the virus sample was mixed with 2× master mix sample buffer containing three fluorescence molecular mass markers, SDS and DTT, and then heated for 10 min at 70 °C. The molecular size-based separation (SDS–PAGE) occurs in the capillary for 30 min at 375V. The gB antibodies were probed for 30 min followed with secondary antibody either anti-rabbit horseradish peroxidase (HRP) or anti-human HRP for 30 min incubation. The chemiluminescence signal was collected with HDR at nine different exposure times (1, 2, 4, 8, 16, 32, 64, 128, 256, and 512 s). The data were analyzed using vendor software, Compas^®^.

### ELISA

The gB protein was immobilized at 2.0 μg/mL in PBS on 96-well Nunc-Immuno plates at 4 °C overnight. Plates were blocked with 3% (vol/vol) nonfat milk in PBS/0.05% Tween-20 for 1 h and then incubated for 1.5 h with serial 3-fold serum starting from 1:40 in 100 μL. Plates were washed after serum incubation and then HRP-conjugated goat anti-human IgG (Southern Biotech) at 1:1000 dilution was added to the plates for 1 h, and then washed. HRP substrate, 3,3/,5,5/ _-_tetramethylbenzidine (Virolabs, Chantilly, VA) was then added in 100 μL volume for 3–5 min, and the reaction was stopped with equal volume of 1 M H_2_SO_4_. The optical density was captured at 450 nm wave length on a plate reader. Endpoint titers were calculated as the highest dilution that was greater than twice the geometric mean of negative controls.

Competition ELISA was used to determine the epitope specificity of gB-specific mAb r210.4 and r350.1 against h272.7, and the method was modified from a procedure published previously.^42^ Briefly, recombinant gB was immobilized at 0.2 μg/mL in PBS on 96-well FluoroNunc plates at 4 °C overnight. Plates were then blocked with 3% nonfat milk in PBS/0.05% Tween-20 for 1 h Rabbit mAbs in titration in PBS was mixed with 0.01 μg/mL of h272.7, and then the mixture was transferred to the plates for 1.5 h. Plates were washed with PBS/0.05% Tween-20 afterwards and then incubated with a HRP-conjugated detection agent, goat anti-human IgG at a 1:500 dilution (Southern Biotech), for 1 h A fluorogenic HRP substrate, 10-acetyl-3,7-dihroxyphenoxazine (ADHP) (Virolabs), was added at 100 μL per well for 3–5 min to generate resorufin and the fluorescent signals with excitation at 531 nm and emission at 595 nm were measured (Victor III; Perkin Elmer). To measure the serum-competition, the immune sera at 1:320 dilution in PBS were mixed with 0.01 μg/mL r272.7 antibody before added to the plate coated with recombinant gB. The plates were then incubated with goat anti-rabbit IgG with HRP conjugate and the signals were developed with ADHP. The percent inhibition by gB/MF59 immune sera was calculated based reduction of signals with the immune sera vs. no immune sera.

### Viral neutralization assay

MRC-5 or ARPE-19 cells were seeded on day 1 at 1.2 × 10^4^ or 1.7 × 10^4^ cells/well, respectively, in 50 μL medium per well in 96-well plates. Heat-inactivated serum or antibody samples in 2-fold serial dilutions were mixed with 6 × 10^4^ pfu/ml of AD169rev-GFP virus in equal volume in the presence or absence of complement. The final dilutions for rabbit and human complement used in the assays were 1:64 and 1:32, respectively.^17^ The mixture was incubated at 37 °C for 1 h and then 50 μL were transferred to MRC-5 or ARPE-19 cells. The plates were cultured for 2 days and cells expressing GFP as a surrogate for HCMV infection were then enumerated using Acumen eX3 laser-scanning fluorescence microplate Cytometer (TTP Lab Tech Ltd, Melbourn, United Kingdom) and Cellista software as previously described.^43^ NT_50_ titers or IC_50_ values were calculated as reciprocal dilution of serum sample or concentration of mAb which achieve 50% reduction in the number of virus infected cells by 4-parameter non-linear curve fittings using Prism^®^ software (GraphPad^®^ Software, San Diego, CA, USA).

### Statistical analysis

All analyses were calculated using Prism^®^ program from GraphPad^®^ (San Diego, CA). The comparison was conducted using unpaired, two-tailed *t*-test. Geometric means and 95% confidence intervals were calculated using algorithms in Prism^®^.

### Data availability

All data supporting figures and presentation are included in this study, and non-published data are available upon request.

## Electronic supplementary material

Figure S1

Figure S2

Figure S3

Figure S4
