# Supplementary material for: Complement enhances in vitro neutralizing potency of antibodies to human cytomegalovirus glycoprotein B (gB) and immune sera induced by gB/MF59 vaccination
Source: NPJ Vaccines. 2017 Dec 14;2:36. doi: 10.1038/s41541-017-0038-0 (PMC5730571; doi:10.1038/s41541-017-0038-0)
Supplement: Supplementary file 1 — Figure S1 [file 41541_2017_38_MOESM1_ESM.pdf]

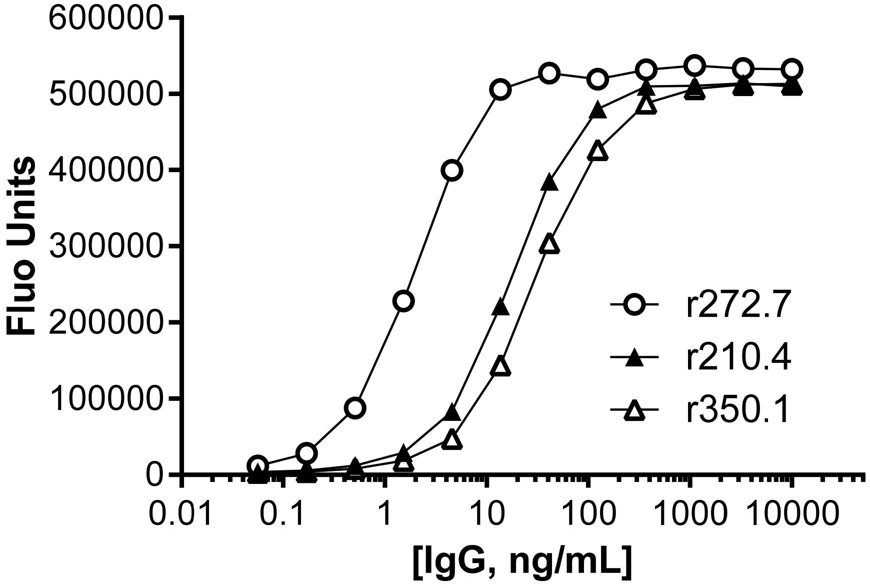

FIG. S1. Rabbit mAbs specific for HCMV gB. Antibodies in titration were measured for their reactivity to recombinant gB immobilized on plates. The concentration to reach 50% fluorescent signals was deduced by four-parameter curve fitting.

Supplemental Figure S1
