# Supplementary material for: Complement enhances in vitro neutralizing potency of antibodies to human cytomegalovirus glycoprotein B (gB) and immune sera induced by gB/MF59 vaccination
Source: NPJ Vaccines. 2017 Dec 14;2:36. doi: 10.1038/s41541-017-0038-0 (PMC5730571; doi:10.1038/s41541-017-0038-0)
Supplement: Supplementary file 2 — Figure S2 [file 41541_2017_38_MOESM2_ESM.pdf]

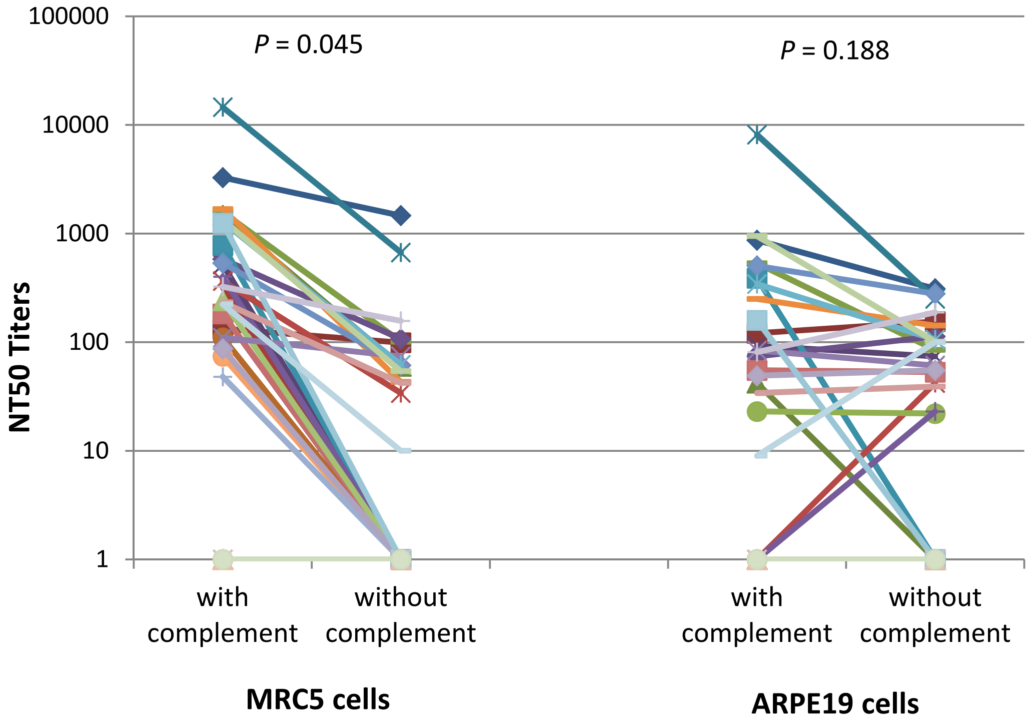

FIG. S2. Complement enhanced neutralizing activity in MRC-5 cells. NT_{50} titers of the peak responses post vaccination (months 6.5 and 7) were compared with or without complement. The p values were from paired, two-tailed *t*-test.

Supplemental Figure S2
