# Supplementary material for: Complement enhances in vitro neutralizing potency of antibodies to human cytomegalovirus glycoprotein B (gB) and immune sera induced by gB/MF59 vaccination
Source: NPJ Vaccines. 2017 Dec 14;2:36. doi: 10.1038/s41541-017-0038-0 (PMC5730571; doi:10.1038/s41541-017-0038-0)
Supplement: Supplementary file 3 — Figure S3 [file 41541_2017_38_MOESM3_ESM.pdf]

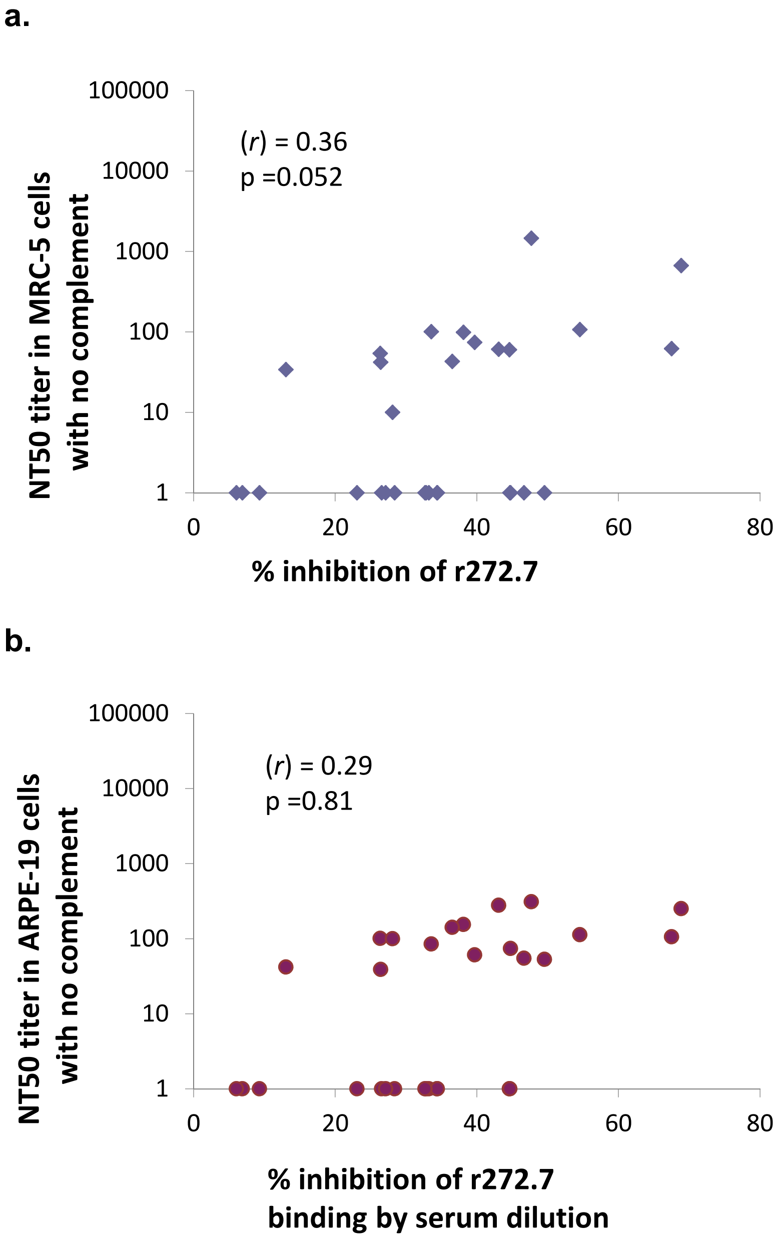

Figure S3. Poor correlation between the neutralizing titers measured with no complement and r272.7-like antibodies in gB/MF59 immune sera. Immune sera obtain at study months 6.5 or 7 were measured for their ability to compete against r272.7 for binding to gB in ELISA. The percent inhibition of r272.7 binding at a fixed serum dilution of 1:320 was plotted vs. NT_{50} titers measured using MRC-5 (A) or ARPE-19 (B) cells with no complement in the assays. (r) represents Pearson correlation coefficient.

Supplemental Figure S3
