# Supplementary material for: Complement enhances in vitro neutralizing potency of antibodies to human cytomegalovirus glycoprotein B (gB) and immune sera induced by gB/MF59 vaccination
Source: NPJ Vaccines. 2017 Dec 14;2:36. doi: 10.1038/s41541-017-0038-0 (PMC5730571; doi:10.1038/s41541-017-0038-0)
Supplement: Supplementary file 4 — Figure S4 [file 41541_2017_38_MOESM4_ESM.pdf]

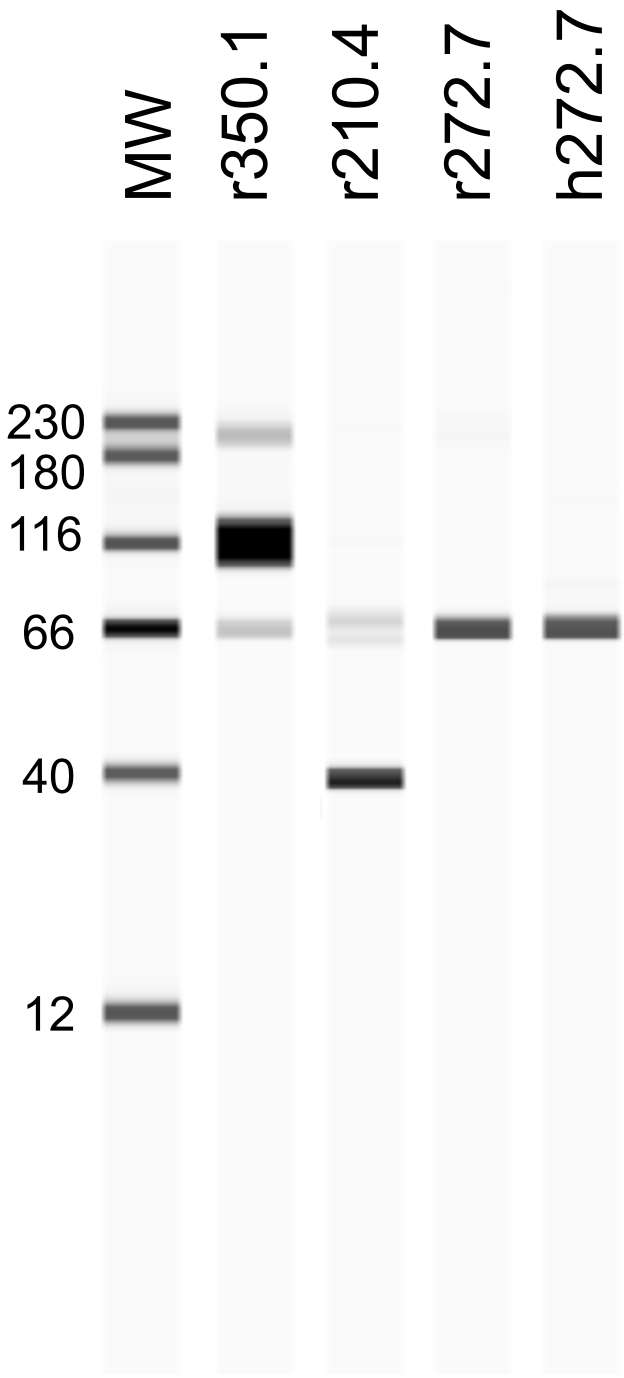

Figure S4. Western blot analysis was conducted in Wes (Protein Simple) system as described in Materials and Methods. Briefly, AD169 virus was denatured and antigens were separated in capillary based on molecular mass. The antigens in the capillary were then blotted with the mAbs as indicated above the blot image, followed by HRP conjugated secondary antibodies. The antigen-specific chemiluminescence signals were recorded and the blot was reconstructed using the software provided by the Vendor. Fluorescent labeled molecular markers are shown on the left

Supplemental Figure S4
